# Evaluation of Process Conditions for Ultrasonic Spray Freeze Drying of Transglutaminase

**DOI:** 10.17113/ftb.58.01.20.6544

**Published:** 2020-03

**Authors:** Hilal Isleroglu, Izzet Turker

**Affiliations:** Tokat Gaziosmanpasa University, Faculty of Engineering and Architecture,; Food Engineering Department, Tasliciftlik Campus, 60150 Tokat, Turkey

**Keywords:** spray freeze drying, ultrasonication, transglutaminase, enzyme activity, flow behaviour, particle morphology

## Abstract

In this study, a commercial transglutaminase enzyme was dried using an ultrasonic spray freeze drying method and the effects of the process conditions were optimized to maximize the final transglutaminase activity. Accordingly, process parameters affecting enzyme activity were selected, such as nozzle frequency (48 and 120 kHz), flow rate (2, 5 and 8 mL/min) and plate temperature for secondary drying (25, 35 and 45 °C). Moreover, the effects of different pH values (pH=2.0 and pH=9.0) and high temperature (80 °C) on enzyme activity, physical properties and particle morphology of transglutaminase were discussed. According to the results, transglutaminase preserved its activity despite ultrasonic spray freeze drying. Sonication enhanced the enzyme activity. Using the desirability function method, the optimum process conditions were determined to be flow rate 3.10 mL/min, plate temperature 45 °C and nozzle frequency 120 kHz. The predicted activity ratio was 1.17, and experimentally obtained ratio was 1.14±0.02. Furthermore, enzyme produced by ultrasonic spray freeze drying had low moisture values (2.92-4.36%) at 8 h of drying. When the morphological structure of the transglutaminase particles produced by ultrasonic spray freeze drying under the optimum conditions was examined, spherical particles with pores on their surfaces were observed. In addition, flow properties of the transglutaminase powders were considered as fair under most conditions according to the Carr index.

## INTRODUCTION

Through recent developments in the food industry, usage of food additives obtained from natural sources to enhance food properties has become popular. Microbial enzymes produced from different strains can be used for the production of more desirable food products or development of novel and unique products. In this respect, protein modification has become an important issue, especially in foods with high protein content. Since proteins are one of the most important components of food products, modification of the food proteins *via* different methods (chemical, physical or enzymatic) can offer novel products and improve their functional properties (*1*). In this regard, transglutaminase (TG) is the most effective enzyme because of its unique properties. Transglutaminase (EC 2.3.2.13) is a transferase catalyzing the acyl transfer reactions between γ-carboxyamide group of protein-bound glutamine residues and primary amines, deamidation of protein-bound glutamines and cross-linking glutamine and lysine peptide residuals (*2*-*4*). It is an extracellular enzyme and generally produced by fermentation of *Streptomyces* strains. TG acts in the pH range of 5.0-8.0 and it is active at 40-70 °C. Also, metal ions (Ca^2+^) or cofactors are not needed for its activation. Because of these unique characteristics, TG is considered a safe food additive for human consumption (*1*, *5*, *6*). As a consequence of its cross-linking properties, a variety of proteins (soy and whey proteins, albumins, myofibrillar proteins) can be suitable substrates for TG (*1*, *2*). Thus, TG can alter the mechanical and textural properties of meat, dairy and bakery products. Many studies have been reported about the successful usage of TG to develop the functional properties of different food products (*7*-*9*).

In the food industry, sonication techniques are generally used for the inactivation of enzymes. Extreme conditions of sonication can cause breakdown of hydrogen bonds and van der Waals interactions in enzyme molecules (*10*). However, ultrasonic applications cannot inactivate all kinds of enzymes. When sonication and ambient conditions are favourable, the biological activity of the enzymes can still be stable. There are several studies in the literature showing that sonication applications have not had negative effects on some enzymes such as γ-glutamyltranspeptidase, lactoperoxidase and chymotrypsin (*11*, *12*). It is also known that some proteins are resistant to sonication and some conformational changes of these proteins may even enhance the enzyme activity (*13*, *14*).

Drying of biological materials such as enzymes is an important topic due to their sensitivity to the process conditions. Lately, spray freeze drying (SFD) has drawn attention for obtaining the powder forms of enzymes used in food industry (*15*). SFD is a process consisting of mainly three steps: *i*) atomization of the bulk solution/liquid, *ii*) freezing of the atomized droplets by cryogenic fluids, and *iii*) drying of frozen particles by sublimation at low temperature and pressure (*15*, *16*). It is asserted that with this method enzymes dried easily and formed fine droplets because of the low temperature during the operation and shorter drying times achieved *via* atomization step (*17*). Different techniques can be used for the atomization of bulk solution, and ultrasonic spraying is the most effective one to generate the fine droplets (*15*, *18*).

When enzymes or protein containing solutions are subjected to drying, stress factors such as freezing and dehydration may affect the biological activity of such products adversely (*19*). Sonner *et al*. (*20*) studied the stability of trypsinogen activity during the ultrasonic spray freeze drying (USFD). They revealed that the activity loss of trypsinogen mainly occurred at the freeze-drying step. Likewise, Yu *et al*. (*21*) investigated the influence of freeze-drying and spray freezing processes on the biological activity of lysozyme and they reported that the drying step decreased the enzyme activity. On the other hand, the adverse effects of the drying step might be eliminated using the ultrasonic nozzles due to positive effects of ultrasonic applications on the enzyme activity. Parallel to this concept, Isleroglu *et al*. (*22*) revealed that the activity of microbial TG was enhanced by ultrasonic atomizing. Furthermore, Ishwarya *et al*. (*15*) stated that the usage of ultrasonication in SFD can enhance the control of particle size distribution and can help production of the porous particles. Hence, D’Addio *et al*. (*18*) carried out USFD successfully to obtain powders of lysozyme with controlled particle size distribution. Although USFD is considered as a suitable method for drying of protein-containing solutions, there are only a small number of studies in the literature revealing the success of it (*20*, *23*). For this reason, the number of studies in which USFD and enzymes are jointly used needs to be increased in order to explain the interactions between them.

The scope of the current study is the optimization of ultrasonic spraying and drying conditions of a commercial TG during USFD to get the highest final enzyme activity. Moreover, physical properties of TG such as bulk and tapped densities, moisture content, water activity and wettability under different conditions were determined. The effect of high temperature and different pH values on each drying was also discussed and particle morphology of the TG powder achieved by the optimization of the USFD was investigated.

## MATERIALS AND METHODS

### Materials

A commercial form of transglutaminase (TG; Tegen 20X; Benosen Gıda San. Dış Tic. Ltd. Şti., Istanbul, Turkey), which was produced by fermentation of *Streptomyces* strains and powdered by spray drying using only maltodextrin as a bulking agent, was used to prepare enzyme solutions. The moisture content and the enzyme activity of the commercial TG powder were (5.4±0.2) % and (4298.8±483.9) U per g protein, respectively. A mass of 15 g of powdered enzyme was dissolved in 60 mL of distilled water, and this solution was prepared freshly before each drying. For determination of enzyme activity, the γ-glutamyl donor substrate of TG (Z-Gln-Gly; Sigma-Aldrich, Merck, Darmstadt, Germany) was used.

### Determination of specific enzyme activity

The specific activity of TG was determined using the hydroxamate method and Bradford protein assay (*24*) and expressed on protein mass basis in U/g. The hydroxamate method was carried out to determine the enzyme activity in U/mL as previously described by Isleroglu *et al*. (*22*). To calculate the specific enzyme activity in U/g, a modified Bradford protein assay was used as follows: first, Bradford solution was prepared. For the preparation of Bradford dye solution, 50 mg brilliant blue G-250 was dissolved in 50 mL pure methanol, then 100 mL 85% (*m/V*) H_3_PO_4_ were added and the mixture was diluted to 1 L with distilled water. The resulting dye solution was stored at 4 °C until use for analysis, and the solution was filtered through Whatman No.1 filter paper before assays. For the analysis, 300 µL of enzyme solution were mixed with 700 µL of dye solution and the absorbance of samples was measured at 595 nm. The protein content of the samples was calculated using bovine serum albumin as a standard and the enzyme activity of samples was determined using the following equation:


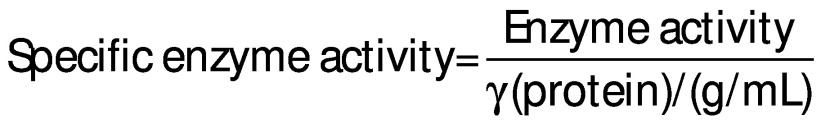


### Ultrasonic spray freeze drying of TG and experimental design

The commercial TG solutions were dried by USFD using different nozzles (SonoTek Inc., Milton, NY, US), an ultrasonic generator (ECHO, SonoTek Inc) and a syringe pump (syringe pump TI; SonoTek Inc). Nozzle frequencies were 48 and 120 kHz, flow rates were 2, 5 and 8 mL/min and plate temperatures of secondary drying phase were set at 25, 35 and 45 °C. The USFD rig was set up according to Isleroglu *et al*. (*22*). A volume of 50 mL of enzyme solution (20%, *m/m*) was atomized and droplets were frozen by the nitrogen vapour. Freezing of the droplets was finished when they all sank into the liquid nitrogen. Under each condition, the frozen particles were dried twice, first for 6 h at 100 Pa and then for 2 h at 1 Pa (Alpha 1-4 LSCplus; Martin Christ Gefriertrocknungsanlagen GmbH, Osterode am Harz, Germany). Plate temperatures were set at the beginning of the second drying phase. Flow rate (X_1_), plate temperature (X_2_) and nozzle frequency (X_3_) were determined as independent variables.

Three-level factorial design was used for the experimental setup (*25*). Flow rate and plate temperatures were selected as numeric factors, nozzle frequency was a categoric factor having two treatments (48 and 120 kHz), and the centre point for each treatment was set at 5.

To determine the total effect of the USFD on TG activity, activity ratio values were used. Initial enzyme activity of the enzyme solutions was determined by measuring the activity of fresh solutions prepared prior to drying. After drying, final activity of powder samples was measured by rehydration of the resulting products to their initial dry matter percentage (20%), matching the fresh solutions used before drying. Activity ratios were calculated using the following equation:


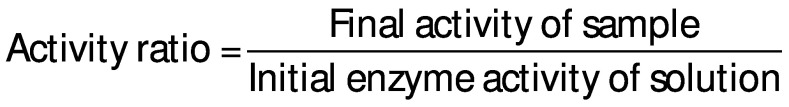


### Investigation of the effect of different pH and temperature on TG

The effect of high temperature on the enzyme activity was investigated at 80 °C instead of 37 °C, as in the standard enzyme activity assay (hydroxamate method). To determine the effect of different pH values on TG activity, buffer solutions (pH=2.0 and pH=9.0) were used instead of pH=6.0 as in standard enzyme activity assay. The results were also defined as activity ratios.

### Physical properties of TG

#### Moisture content

All samples were analyzed by an infrared moisture analyzer (MOC63u; Shimadzu, Tokyo, Japan) to determine the moisture content. The analyses were carried out at 90 °C using the device’s automatic mode. All analyses were done in duplicate.

#### Water activity

The water activity measurements of the powder samples were carried out with a water activity measurement device (3TE; AquaLab Decagon Devices Inc., Pullman, WA, US) and all measurements were done in parallel.

#### Bulk and tapped densities and flow behaviour

The masses of the powder samples and the volume of the samples of the same mass were measured to determine the bulk density (*ρ*_b_). First, approx. 2 g of sample were weighed and the exact mass was recorded. Then, the sample was poured into a measuring cylinder (25 mL) and the volume was determined. *ρ*_b_ values were calculated by dividing the mass of the powders by their volumes. After the volume values were determined for *ρ*_b_, the measuring cylinder was tapped 100-150 times until a steady volume was reached to calculate tapped density (*ρ*_t_). The *ρ*_t_ of the samples was calculated by dividing the mass of the samples by their tapped volume (*22*).

Carr index (CI) and Hausner ratio (HR) values were also calculated for bulk and tapped densities to determine the flow behaviour of the powder samples (*26*, *27*). The following equations were used to calculate CI and HR, respectively:


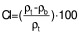


and


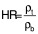


#### Wettability

Wettability assay of the powder samples was carried out according to the method described by Isleroglu *et al*. (*22*). Samples of 0.1 g were analyzed for every run and the wettability values were identified as the time needed for the powders to get totally wet. All analyses were done in duplicate.

#### Particle morphology

Particle morphology analysis was carried out by scanning electron microscopy of the sample obtained under the determined optimum process conditions (EVO LS 10; Carl Zeiss, Microscopy GmbH, Jena, Germany). Aluminium specimen stubs with conductive carbon- adhesive tape on their surfaces were covered with samples coated with gold (200 s). Images were taken at different magnifications. The analyses were done under a 1.30·10^-2^ Pa vacuum and accelerating voltage of 20 kV.

### Statistical analysis

Experimental data obtained from the design were fitted to a second-order polynomial model and the desirability function method was used to determine the optimum process conditions to ensure the highest activity ratio. Analysis of variance was used to determine the significant variables at a confidence level of 95%, and the p-values of lack of fit were expected to be over 0.05. Verification of the optimum point was carried out by one sample *t*-test. After investigating the effects of different pH and temperature on TG, the data were analyzed by Duncan’s multiple range test (*28*).

## RESULTS AND DISCUSSION

The activity ratio values ranged between 0.76 and 1.31 (Table 1). One can observe that under most conditions the final activity was higher than the initial activity of the enzyme solution. Considering that the protein content of the samples could not be changed by the USFD process, it can be assumed that the activity (U/mL) of the enzyme increased under most of the process conditions. It is known that the stability of enzymes under sonication conditions is unique and specific, as the conformational structures of all kinds of enzymes are different (*14*). Isleroglu *et al.* (*22*) found the positive effect of the ultrasound treatment on TG activity, *i.e*. the activity after sonication and before drying was higher than the initial activity of the enzyme (activity ratio between 1.02 and 1.31). Furthermore, Isleroglu *et al*. (*29*) observed the same results for microencapsulated TG. According to their results, the enzyme activity increased during the atomization and freezing stage, which was not related to the coating type or other process conditions. These studies showed that atomization and freezing did not harm the TG; conversely, its activity was slightly enhanced.

**Table 1:** Experimental design with variable flow rate (*Q*), plate temperature (*t*) and nozzle frequency (*ν*) and the obtained activity ratio values

X_1_ *Q*/(mL/min)	X_2_ *t*(plate)/°C	X_3_ *ν*(nozzle)/kHz	Activity ratio
2	25	48	0.96±0.03
5	25	48	1.01±0.01
8	25	48	1.04±0.04
2	35	48	1.03±0.00
5	35	48	1.10±0.00
5	35	48	1.13±0.00
5	35	48	1.02±0.01
5	35	48	1.01±0.01
5	35	48	1.02±0.02
8	35	48	1.17±0.02
2	45	48	0.91±0.00
5	45	48	1.12±0.00
8	45	48	1.06±0.00
2	25	120	0.88±0.01
5	25	120	1.09±0.00
8	25	120	0.76±0.01
2	35	120	0.97±0.01
5	35	120	1.31±0.06
5	35	120	1.06±0.01
5	35	120	1.14±0.01
5	35	120	1.08±0.01
5	35	120	1.05±0.01
8	35	120	0.87±0.01
2	45	120	1.22±0.03
5	45	120	1.12±0.01
8	45	120	0.82±0.00

Many studies have shown the inhibitory effect of sonication on different kinds of enzymes. Raviyan *et al*. (*30*) studied the thermal and thermosonication inactivation kinetics of pectin methylesterase in tomatoes, revealing that the sonication process positively affected the inhibition of the enzyme. Likewise, Tian *et al*. (*31*) revealed that the activity of trypsin in an aqueous medium decreased with an increase in ultrasound power (20 kHz, 100-500 W). Although sonication is mainly used for inactivation of enzymes as described in literature, there are several studies showing positive effects or at least inhibiting effects of ultrasound treatments on enzyme activity. Mañas *et al*. (*32*) investigated the effect of ultrasound on egg white lysozyme and found that a 15-minute application of ultrasound at 20 kHz frequency did not affect enzyme activity. Apar *et al*. (*33*) also demonstrated that α-amylases produced from *Bacillus* species were not inhibited by sonication of 20 kHz. Moreover, Gębicka and Gębicki (*34*) determined that catalase was not inactivated under the conditions of 22 kHz frequency and 5 °C of ambient temperature. Similar to our study, Froment *et al*. (*35*) determined a slight transient enzyme activation for human butyrylcholinesterase at varying density input, using sonication frequency of 20 kHz. They concluded that the small changes in the catalytic activity might be caused by slight ultrasound-induced conformational changes affecting the active site reactivity. In our study, it is thought that the active TG site was rearranged by ultrasonic forces, which might lead to a positive effect on the enzyme activity when suitable process conditions are applied. As stated by some researchers, ultrasound treatments at proper frequencies and intensity may cause an increase in enzyme activity through physical and biochemical effects (*10*).

For USFD, flow rate and nozzle frequency are the most important parameters affecting the success of the operation (*15*). Moreover, drying time is an important factor that can lead to critical decreases in the enzyme activity (*20*). In our previous study, in which microbial TG was used, it was determined that atomization did not affect the TG activity negatively; however, the activity decreased at the drying step drastically (*22*). In this study, TG activity was thought to be enhanced by ultrasonic atomization. This increase in the enzyme activity at the atomization step might have defeated the negative effects of drying, such as dehydration stress on enzyme activity. Under these circumstances, the effect of sonication was prominently seen.

The mathematical model that expresses the relationship between the process variables was formed by multiple linear regression analysis. For this purpose, linear effect terms for each variable were first used. Then, quadratic and interaction effect terms were added to the model, and the increase in the sum of squares and lack of fit values were analyzed (Table 2). The model was found to be statistically significant at the confidence level of 95% (p<0.05). According to the data from Table 2, the linear effects of different parameters did not affect the activity ratio significantly (p>0.05). Interaction of flow rate and nozzle frequency affected the activity ratio significantly (p<0.05); however, the enzyme activity was not affected by the plate temperature (p>0.05) according to the used model.

**Table 2:** Statistical results for the effects of process parameters on activity ratio

Source	DF	Activity ratio							
pH=6.0 (main condition)		pH=2.0		pH=9.0		*t*=80 °C	
Sum of squares	p-value	Sum of squares	p-value	Sum of squares	p-value	Sum of squares	p-value
Model	8	0.22	0.0325	0.016	0.9496	0.042	0.1727	0.046	0.1126
X_1_	1	5.54·10^-3^	0.4541	2.35·10^-3^	0.5481	1.67·10^-3^	0.4753	0.011	0.0724
X_2_	1	0.022	0.1487	3.26·10^-4^	0.8220	4.53·10^-5^	0.9056	1.61·10^-3^	0.4669
X_3_	1	1.84·10^-3^	0.6648	3.27·10^-4^	0.8217	0.013	0.0598	0.016	0.0300
X_1_X_2_	1	5.51·10^-3^	0.4553	2.39·10^-3^	0.5445	4.71·10^-3^	0.2360	3.67·10^-4^	0.7273
X_1_X_3_	1	0.082	0.0091	6.74·10^-3^	0.3139	9.56·10^-3^	0.0982	7.56·10^-3^	0.1258
X_2_X_3_	1	0.010	0.3067	2.00·10^-3^	0.5790	8.35·10^-4^	0.6117	1.99·10^-3^	0.4205
X_1_^2^	1	0.059	0.0233	2.79·10^-4^	0.8353	0.012	0.0713	2.88·10^-3^	0.3341
X_2_^2^	1	4.90·10^-3^	0.4810	1.61·10^-3^	0.6188	5.23 ·10^-3^	0.2131	7.07·10^-3^	0.1379
Residual	17	0.16		0.11		0.053		0.050	
Lack of fit	9	0.10	0.2876	0.072	0.1950	0.035	0.2436	0.039	0.0531
Pure error	8	0.060		0.034		0.019		0.010	
Total	25	0.38		0.12		0.095		0.096	

DF=degree of freedom, X_1_=flow rate/(mL/min), X_2_=plate temperature/°C, X_3_=nozzle frequency/kHz

**Fig. 1:**
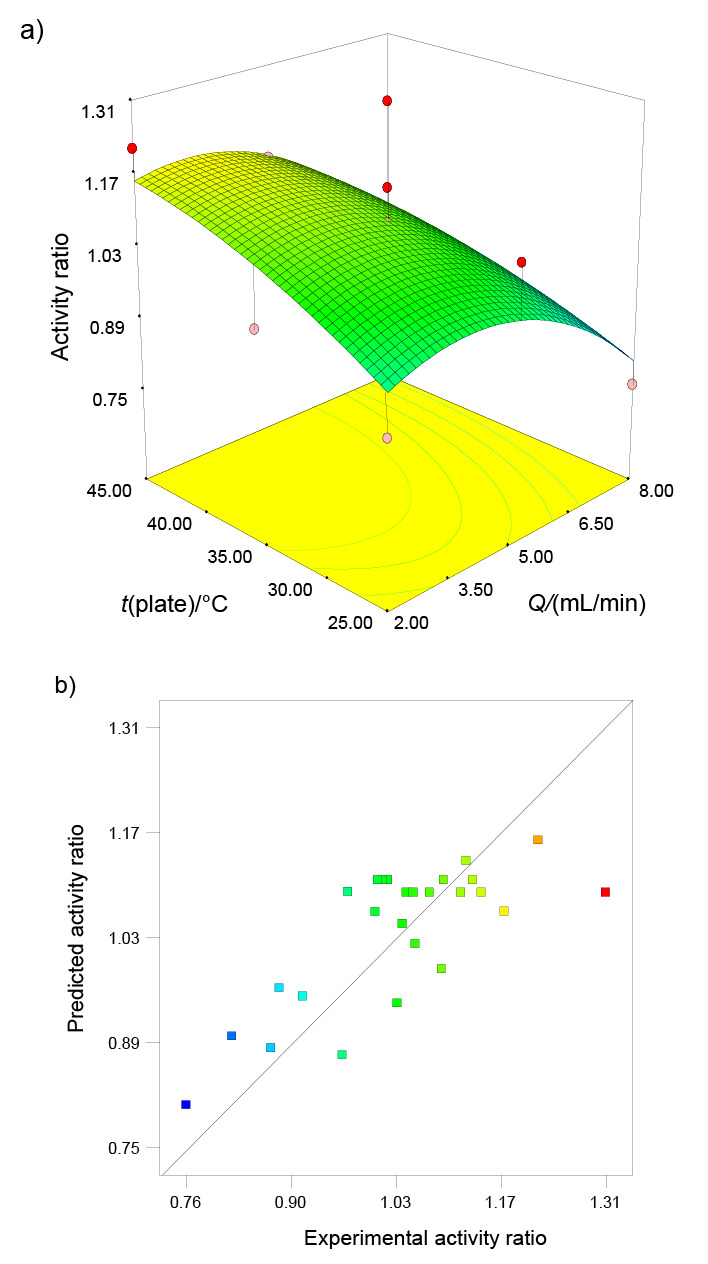
Effects of plate temperature (*t*) and flow rate (*Q*) on the activity ratio: a) 3D response surface graph for 120 kHz, and b) the relation between the experimental and predicted values

The 3D response surface graph and contour lines are shown in Fig. 1a for 120 kHz nozzle frequency when the activity ratio was chosen as a response. The circular form of the lines demonstrates that the interaction between the flow rate and plate temperature is not significant as previously shown by the ANOVA results (Table 2). Furthermore, the linear trend of the plate temperature (X_2_) axis showed that plate temperature did not have a significant impact on activity ratio (p>0.05). The second-order polynomial model (obtained from regression analysis for the activity ratio used for the optimization study) is given in Eq. 5 using coded variables. The relationship between the values estimated from Eq. 5 and the experimental values for the activity ratio is shown in Fig. 1b. It can be seen that the model’s activity ratio estimates were consistent with experimental data.


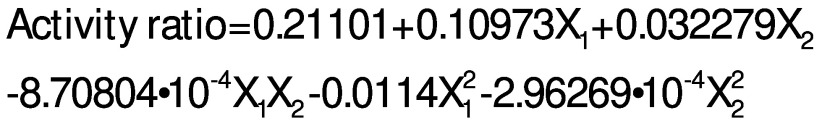


**Fig. 2:**
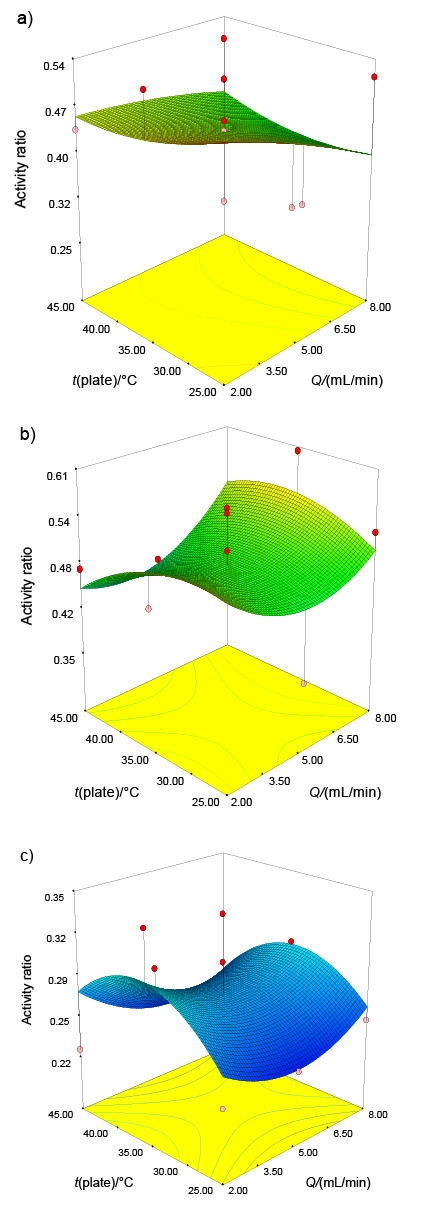
Effects of plate temperature (*t*) and flow rate (*Q*) on the activity ratio at: a) pH=2.0, b) pH=9.0, and c) *t*=80 °C

TG is an enzyme that can be used in various food systems with different pH values. At different pH values, the activity changes of TG are an important topic affecting its usage in the process. To determine the process conditions on TG activity at different pH values, buffer solutions of pH=2.0 and pH=9.0 were used instead of Tris buffer solution pH=6, as described in the activity assay (*22*). At pH=2.0, activity ratios ranged between 0.25 and 0.53 (Fig. 2a). Likewise, TG activity was also inhibited at pH=9.0 and activity ratios were in the range of 0.34-0.60 (Fig. 2b). TG activity decreased dramatically under all conditions; however, at least 25% of the activity was preserved. Isleroglu *et al*. (*22*) demonstrated that minimum and maximum relative activity values ranged between 27 and 67% at pH=2.0 and pH=9.0. However, Cui *et al*. (*36*) demonstrated that microbial TG lost all activity at lower pH. In this study, none of the process conditions statistically affected the activity ratios of different pH values (p>0.05) (Table 2). Nevertheless, the effect of sonication is thought to be an important factor because of causing conformational changes on the surface of enzyme molecules, which render TG more stable at different pH levels.

Temperature is another substantial parameter for the TG activity. The effect of high temperature on sonicated TG was investigated to reveal the resistance of the enzyme activity. Similar to the changes at different pH, the activity ratios decreased more sharply at high temperature at both nozzle frequencies. Nevertheless, at 80 °C for 1 h of incubation, the enzyme still had an activity ratio of at least 0.23 (Fig. 2c). When ANOVA was applied for model parameter effects on activity ratios at high temperature, only nozzle frequency had a significant statistical effect (p<0.05) (Table 2). The greater resistance of TG produced with a 120 kHz nozzle frequency at 80 °C than that of a 48 kHz can be explained by a stronger structural conformational change due to higher frequency.

For the optimization study, activity ratio values were used to determine the effects of independent variables on TG activity. The desirability function method was chosen to obtain the highest activity ratio at different conditions; namely, flow rate, plate temperature and nozzle frequency. The optimum point was determined at flow rate 3.10 mL/min, plate temperature 45 °C and nozzle frequency 120 kHz with a desirability of 0.76. Calculated activity ratio was 1.17, and validation of estimated optimum point prediction was carried out under the selected conditions. The experimental result (activity ratio) at the optimum point was 1.14±0.02. Experimental results did not differ statistically when compared with the values predicted by the model according to the paired *t*-test results (p>0.05).

**Fig. 3:**
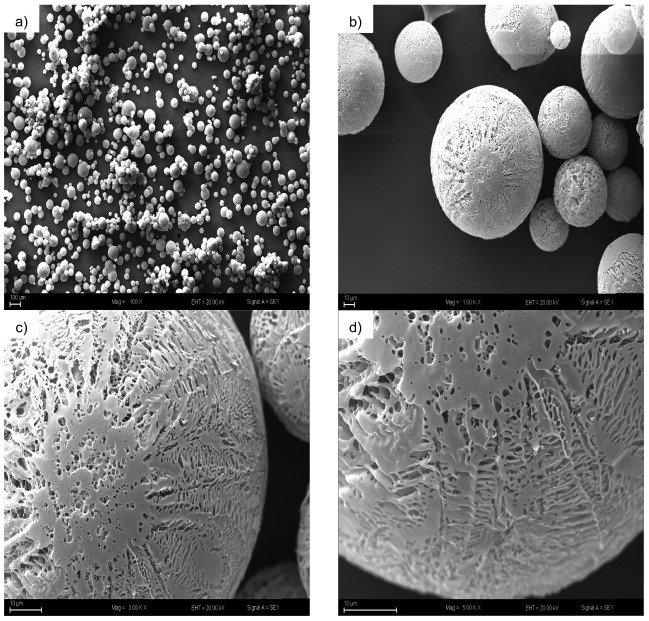
Particle morphology of the obtained transglutaminase by scanning electron microscopy at different magnifications: a) 100×, b) 1000×, c) 3000× and d) 5000×

The physical properties of TG powders under each USFD condition were also determined (Table 3). Moisture content, which is one of the most important parameters for powdered products, can especially affect the storage stability of biological materials. Water activity is considered as a key parameter for storage, like moisture content, and these parameters should be controlled carefully (*37*). The moisture values ranged between 2.92 and 4.36%, and all samples showed water activity lower than 0.035, which is under the limit of our water activity measurement device. The obtained results were very promising for an enzyme powder, which means that if this powder form can be stored carefully, the enzyme can remain active for a very long time. Samples produced under any condition in this study might have longer shelf-life with reduced possibility of oxidation and microbial growth because of their very low water activity (*38*). It is also assumed that the generation of fine crystal formation at the rapid freezing step of the USFD may lead to the production of porous particles, resulting in lower moisture content and water activity (*17*). When individual particles have a porous surface upon atomization and freezing, drying time can get shorter and moisture values can be lower (*15*). Table 4 shows the effects of the process parameters and their influence on the physical properties of TG powder samples. According to the results, plate temperatures had the most statistically significant effect on moisture values (p<0.05), and when the plate temperature was increased, the moisture values of powdered TG decreased. In this respect, the highest plate temperature selected in the study (45 °C) did not have an impact on the activity ratio (p>0.05), and powder products with low moisture content were obtained. Bulk and tapped densities of the powder products are important parameters for packaging, transportation and storage (*39*, *40*). In this study, bulk and tapped densities were calculated in the range of 195-257 and 268-334 kg/m^3^, respectively (Table 3). However, it was determined that none of the process parameters or their interactions statistically affected bulk or tapped density (p>0.05) (Table 4). Wettability is considered an important reconstitution property, and wettability of powder products is mainly affected by their drying method (*41*). Our results showed that wettability values obtained at a 120 kHz nozzle frequency were lower, and this phenomenon might be related to the smaller particle size of the samples regarding the higher sonication frequency (Table 3). The inverse relation between the particle size and atomization frequency has been previously described in different studies (*22*, *42*). ANOVA results showed that only nozzle frequency statistically affected wettability (Table 4). The flowability of powders and their flow behaviour under different conditions are very important for processing and transportation operations (*43*). In this study, flowability of the powders was defined by Carr index and Hausner ratio. The critical compressibility value between free-flowing (granular) and non-free-flowing (powder) is about 20-25%. Most of the samples showed ‘fair’ flowability, while some of them exhibited ‘good’ flowability in terms of Carr’s classification (*26*). The linear effect of flow rate and plate temperature had a significant impact (p<0.05) on Carr index and Hausner ratio values, whereas nozzle frequency and interactions of the independent variables did not (p>0.05) (Table 4). The SEM images of TG produced at the optimum conditions (Fig. 3) show the spherical shape of the sample and the fair flowability of samples can be explained by the generation of the spherical particles. These tend to decrease the cohesive forces, resulting in an increase in flowing ability. Similar results were obtained in other studies on spray freeze drying (*44*).

**Table 3:** Physical properties of transglutaminase (TG) under different drying conditions of nozzle frequency (*ν*), plate temperature (*t*) and flow rate (*Q*)

*ν*(nozzle) /kHz	*t*(plate) /°C	*Q*/(mL/min)	*w*(moisture)/%	*ρ*(bulk)/(kg/m^3^)	*ρ*(tapped)/(kg/m^3^)	Wettability/s	CI/%	HR
48	25	2	(3.3±0.2)^a^	(205.5±1.4)^a^	(298.6±3.8)^ab^	(4.21±0.04)^ab^	(31.2±0.4)^abc^	(1.45±0.01)^bc^
		5	(3.9±0.5)^a^	(219.3±1.7)^a^	(314.0±4.3)^ab^	(6.7±0.3)^ab^	(30.1±0.4)^bc^	(1.43±0.01)^bc^
		8	(3.6±0.2)^a^	(204.5±0.3)^a^	(249.2±0.3)^b^	(7.8±0.3)^a^	(17.95±0.00)^e^	(1.22±0.00)^e^
	35	2	(3.5±0.2)^a^	(195.9±1.2)^a^	(267.7±1.7)^ab^	(3.8±0.3)^b^	(26.83±0.00)^bcd^	(1.37±0.00)^bcd^
		5	(3.1±0.2)^a^	(216.5±25.4)^a^	(301.3±46.2)^ab^	(6.2±2.0)^ab^	(27.8±3.6)^bcd^	(1.39±0.07)^bcd^
		8	(3.6±0.2)^a^	(205.9±3.9)^a^	(278.4±9.0)^ab^	(6.7±0.30)^ab^	(26.0±1.0)^cd^	(1.35±0.02)^cd^
	45	2	(3.7±0.1) ^a^	(200.8±0.7)^a^	(317.3±2.9)^ab^	(4.6±0.2)^ab^	(36.7±0.8)^a^	(1.58±0.02)^a^
		5	(2.92±0.04)^a^	(209.5±2.1)^a^	(272.4±4.8)^ab^	(5.6±0.1)^ab^	(23.1±0.6)^de^	(1.30±0.01)^de^
		8	(3.2±0.1)^a^	(225.6±1.9)^a^	(333.6±1.9)^a^	(5.8±0.3)^ab^	(32.4±1.0)^ab^	(1.48±0.02)^ab^
120	25	2	(3.7±0.2)^xyz^	(211.9±1.0)^z^	(307.9±0.3)^z^	(4.1±0.1)^yz^	(31.2±0.4)^z^	(1.45±0.01)^yz^
		5	(3.7±0.2)^xyz^	(256.7±3.3)^z^	(320.8±0.8)^z^	(4.9±0.2)^yz^	(20.0±1.2)^x^	(1.25±0.02)^x^
		8	(4.4±0.2)^z^	(202.4±1.6)^z^	(258.1±7.1)^z^	(3.4±0.2)^y^	(21.5±1.5)^xy^	(1.28±0.02)^xy^
	35	2	(3.5±0.3)^xyz^	(202.4±0.4)^z^	(290.8±2.2)^z^	(4.1±0.1)^yz^	(30.4±0.4)^yz^	(1.44±0.01)^xyz^
		5	(3.2±0.2)^x^	(228.2±56.7)^z^	(313.6±61.0)^z^	(5.5±1.0)^z^	(28.0±5.8)^xyz^	(1.40±0.01)^xyz^
		8	(4.0±0.1)^yz^	(220.2±1.0)^z^	(298.9±1.3)^z^	(3.7±0.3)^y^	(26.32±0.00)^xyz^	(1.36±0.00)^xyz^
	45	2	(3.5±0.2)^xy^	(216.0±5.2)^z^	(326.9±14.9)^z^	(3.4±0.2)^y^	(33.9±1.4)^z^	(1.51±0.03)^z^
		5	(3.09±0.07)^x^	(200.10±0.00)^z^	(294.7±1.7)^z^	(4.6±0.2)^yz^	(32.1±0.4)^z^	(1.47±0.01)^z^
		8	(3.37±0.07)^xy^	(231.4±0.7)^z^	(323.9±1.0)^z^	(3.7±0.2)^y^	(28.57±0.00)^xyz^	(1.40±0.00)^xyz^

^a–e^Mean values with different letters in superscript within a column at the same flow rate are significantly different (p<0.05) for *ν*(nozzle)=48 kHz

^x–z^Mean values with different letters in superscript within a column at the same flow rate are significantly different (p<0.05) for *ν*(nozzle)=120 kHz

*Water activity values <0.035 are not given in the table

CI=Carr index, HR=Hausner ratio

**Table 4:** Statistical results for the effects of model parameters on physical properties

Source	DF	*w*(moisture) /%	*ρ*(bulk) /(kg/m^3^)	*ρ*(tapped) /(kg/m^3^)	Wettability/s	CI/%	HR
p-value					
Model	8	0.0010	0.9629	0.9348	0.0393	0.2167	0.2668
X_1_	1	0.1969	0.6126	0.6488	0.1066	0.0296	0.0337
X_2_	1	0.0026	0.8812	0.4166	0.3982	0.0411	0.0481
X_3_	1	0.1764	0.4278	0.4847	0.0143	0.9746	0.9639
X_1_X_2_	1	0.0251	0.5848	0.3543	0.7036	0.3205	0.4457
X_1_X_3_	1	0.2299	0.9290	0.8791	0.0569	0.9656	0.9491
X_2_X_3_	1	0.3461	0.7900	0.9851	0.6480	0.5770	0.6333
X_1_^2^	1	0.0012	0.3611	0.5271	0.0483	0.5730	0.5986
X_2_^2^	1	0.2997	0.8585	0.7784	0.7172	0.9792	0.8736
Residual	17						
Lack of fit	9	0.2007	0.9977	0.9774	0.9943	0.6493	0.7002
Pure error	8						

DF=degree of freedom, X_1_=flow rate/(mL/min), X_2_=plate temperature/°C, X_3_=nozzle frequency/kHz, CI=Carr index, HR=Hausner ratio

The observation of the microstructure of spray freeze dried products is important to determine whether the collapse phenomenon is absent or not, which can be a signature of successful drying operation (*17*). In our study, SEM images of TG produced at the optimum conditions were taken at magnifications of 100, 1000, 3000 and 5000× (Fig. 3). At 100× magnification, tiny spherical particles having moderate particle size distribution were observed (Fig. 3a). When a 1000× magnified image was investigated, spherical particles with pores on their surface were clearly observable (Fig. 3b). The same spherical particles were observable 3 times closer at 3000× magnification (see Fig. 3c), and it was evident that the spherical particles had small fine pores on their surfaces depending on the sublimation of ice crystals. In Fig. 3d, 5000× magnification was used and the fine pores were observed in much better detail, indicating that no collapse occurred during drying. The fine and small pores are thought to have formed because of fast freezing rates and pore sizes much smaller than of the freeze-dried products (*17*). Our findings were consistent with spray-freeze studies found in literature (*17*, *45*). The low moisture values of the USFD samples in our study might be strongly related with particle morphology. The porous structure of TG particles produced by USFD might also have an impact on the low wettability values of the samples (Table 3). It is thought that TG particles rapidly dissolve due to the fast infusion of water through micropores. Rogers *et al*. (*46*) reported similar findings in their characterization study of milk powders produced by SFD. Moreover, the TG activity might be enhanced by configuration of the surface proteins of the enzyme, followed by generation of the particles having micropores on their surface due to fast freezing and sublimation.

## CONCLUSION

In this study, the ultrasonic spray freeze drying (USFD) conditions for obtaining transglutaminase (TG) powder were optimized to obtain the highest possible activity ratio. Moreover, physical properties of the powders produced using USFD were determined, and the process parameters affecting their physical properties were considered. The effects of the process on the activity ratios at different pH values and at high temperature were also investigated. Additionally, SEM images of the powdered TG were taken in order to observe the microstructure of the particles. The results showed that the USFD did not negatively affect the biological activity of TG. Furthermore, TG activity increased after the whole process. Low moisture content and fair flowability of TG samples were also obtained by USFD. Moreover, the spherical and porous surface of USFD powders were shown by SEM images. The findings of this study indicate that TG powder can be produced by the USFD method while increasing its activity. Even though USFD is an expensive technique for drying, it can be a feasible way to produce TG in powder form for large-scale production in the long term because of its low moisture content and the resulting higher activity of the enzyme powders.
